# *Burkholderia pseudomallei* peri-prosthetic infection following medial malleolar internal fixation: a case report

**DOI:** 10.1186/s12879-020-04967-y

**Published:** 2020-03-19

**Authors:** Umesh Jayarajah, Arulprashanth Arulanantham, Vimaleswaran Koculen, Chamikara Palkumbura, Aadil Faleel, Rukshan Sooriyarachchi

**Affiliations:** Department of Orthopaedics and Trauma, National Hospital of Sri Lanka, Colombo, Sri Lanka

**Keywords:** Melioidosis, *Burkholderia pseudomallei*, Peri-prosthetic infection, Medial malleolar internal fixation, Screw fixation, Chronic osteomyelitis, Septic arthritis, Case report

## Abstract

**Background:**

Melioidosis-associated peri-prosthetic infection is extremely rare. To date, melioidosis associated septic arthritis of the ankle joint following a medial malleolar internal fixation has not been reported.

**Case presentation:**

We describe a 49-year-old male with a history of long standing diabetes who presented with fever, constitutional symptoms and right ankle pain for 1 week. Ten years ago, he underwent a medial malleolar screw fixation following a traumatic closed fracture. His initial right ankle radiographs showed no evidence of osteomyelitis. He underwent a wound debridement and washout of the right ankle joint. The peripheral blood and pus from the ankle joint was culture positive for *Burkholderia pseudomallei* with very high antibody titres. His subsequent radiographs showed features of chronic osteomyelitis. He was treated with a prolonged course of antibiotics and repeated wound debridement. At follow up after 6 months, he had no clinical features of recurrent infection.

**Conclusions:**

Melioidosis should be entertained in the differential diagnosis of peri-prosthetic infections in high risk patients.

## Background

Melioidosis is endemic in many tropical countries [1]. The infection is caused by the gram-negative bacterium *Burkholderia pseudomallei* [1]. The bacterium exists in the soil and water and is notorious in causing multiple abscesses in the lung and solid organs in susceptible individuals [2].

Melioidosis is associated with a wide spectrum of clinical manifestations ranging from isolated cutaneous lesions to systemic illness and death [3]. Respiratory and cardiovascular manifestations are the commonest. Pneumonia is the presenting illness in about 50% of cases and other respiratory manifestations include pulmonary abscess and pleuritis [3]. Cardiovascular manifestations include bacteraemia without an evident focus, pericarditis and mycotic aneurysms [2, 3]. Disseminated infections may occur with involvement of the gastrointestinal, urinary, musculoskeletal and central nervous systems as previously reported. Skin and soft tissue involvement, which may be isolated, generally occurs after direct inoculation of the organism. Other rare manifestations include mastitis and lymphadenitis [2, 3].

The most prevalent predisposing factor for melioidosis is diabetes mellitus which is seen in more than half the population [3]. Other risk factors include male sex, age more than 45 years, exposure to soil or water, excess consumption of alcohol, chronic organ impairment involving the liver, lungs and/or kidneys and haematological conditions such as thalassaemia [3]. Long-term steroids and immunosuppression may also predispose to infection. However, a considerable proportion (about 20% of adult patients) have no identifiable risk factors [3].

The occurrence of musculoskeletal infection is infrequently seen and includes osteomyelitis, soft tissue infection and septic arthritis [4]. Melioidosis associated peri-prosthetic infection is extremely rare. To date, melioidosis associated infection of the ankle joint following an internal fixation has not been reported. We describe a man with long standing history of diabetes who developed melioidosis associated peri-prosthetic infection over the ankle joint.

## Case presentation

A 49-year-old Sri Lankan man with a history of long standing type 2 diabetes mellitus and hypertension, presented with fever, loss of appetite and generalised body aches for 1 week duration. He had a past history of right medial malleolar screw fixation 10 years ago following a traumatic uncomplicated closed fracture. He was diagnosed with type 2 diabetes mellitus 20 years ago; however, he was lost to follow-up with poor glycaemic control demonstrated by a HbA1c on admission of 8.5%. He was a non-smoker and consumed alcohol occasionally. He did not have any history of chronic lung disease or steroid use. There was no recent history of trauma or break in skin integrity and there were no cutaneous lesions or ulcers evident on examination. However, he was a tractor driver and had a history of exposure to mud in paddy fields. Due to the presence of fever with considerable myalgia and exposure to mud in paddy fields, he was initially suspected to have leptospirosis and was started on intravenous ceftriaxone at the local hospital. Four days later, he developed right ankle swelling and discolouration and was transferred to the tertiary care centre for further management.

**Fig. 1:**
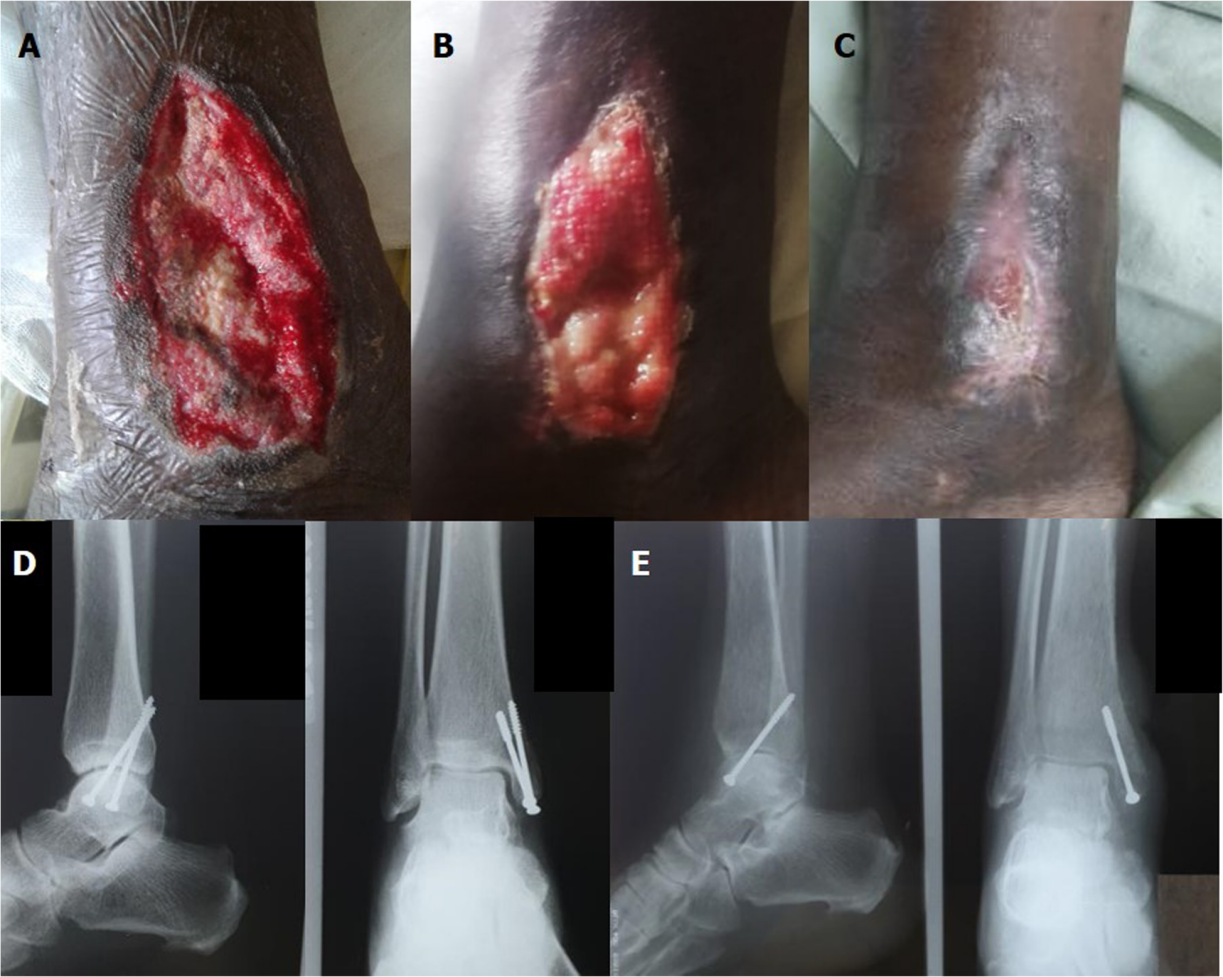
**a**, **b** and **c**: Right medial malleolar wound after repeated debridement at 1 week, 3 weeks and 5 weeks. **d** and **e**: Lateral and anteroposterior views of right ankle radiograph on admission and at 2 months after initiating antibiotics

On admission, he was febrile (103 °F) and tachycardic with a pulse rate of 104 / min. His other haemodynamic parameters were normal with a blood pressure of 140/80 mmHg and was mildly dehydrated with a urine output of 0.5 ml/kg/hour. He was not haemodynamically compromised due to sepsis and his urine output improved with fluid resuscitation. He had a white blood cell count of 15 × 10^3^/uL with a neutrophil leucocytosis; C-reactive protein was 190 mg/dl and erythrocyte sedimentation rate was 86 mm/hour. His platelet count was 148 × 10^3^/uL. His liver enzymes were mildly elevated and rest of the basic biochemistry was unremarkable (alanine transaminase: 252 U/L, aspartate transaminase: 222 U/L, serum creatine: 0.46 mg/dl). The X-ray of the right ankle revealed a healed fracture with two screws which were in position. There was no evidence of osteomyelitis (Fig. 1).

He underwent a wound debridement of the right ankle and the necrotic skin and subcutaneous tissue were excised. The pus collection in the subcutaneous tissue plane was removed. Aspiration of the ankle joint revealed purulent fluid. Therefore, an arthrotomy and washout was done. One loosely fitted screw was removed and the wound was left open. Removal of the second screw was considered but this procedure was abandoned due to technical difficulties and concern about iatrogenically causing a fracture in the process.

**Fig. 2:**
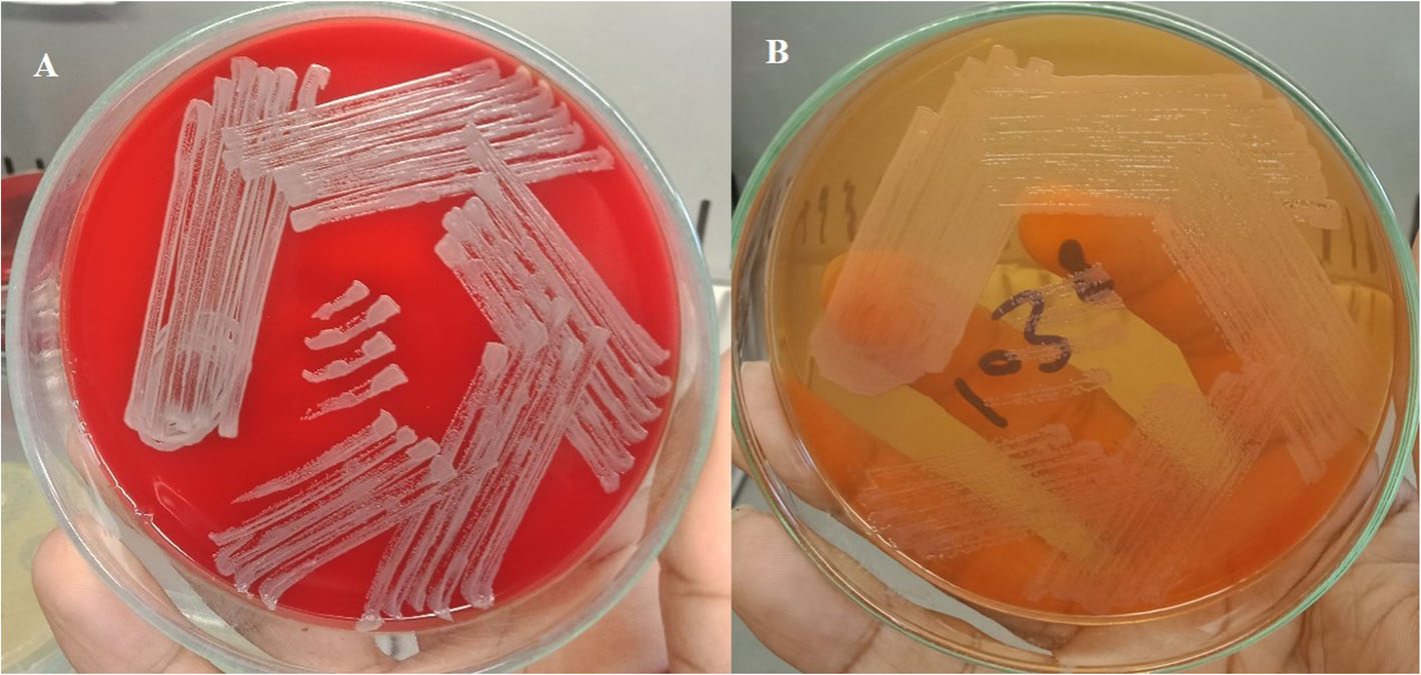
Culture showed non-lactose fermenting colonies with a metallic sheen were isolated on blood agar (**a**) and MacConkey agar (**b**)

The pus culture and the peripheral blood culture was positive for *Burkholderia pseudomallei* sensitive to meropenem and co-trimoxazole. Direct smear showed gram-negative bacilli with bipolar appearance. Non-lactose fermenting colonies with a metallic sheen were isolated on blood and MacConkey agar, and *Burkholderia pseudomallei* species were isolated using the latex agglutination test (Fig. 2). The BD PHOENIX (Becton Dickinson Diagnostic Systems, Sparks, Md.) automated microbiology system for direct identification also confirmed the presence of *Burkholderia pseudomallei*.

His melioidosis antibody titre using the indirect haemagglutination assay was > 10,240. He was empirically managed with intravenous ceftriaxone 1 g 12 hourly and metronidazole 500 mg 8 hourly for 7 days. This was because Leptospirosis or anaerobic infection of the lower limb was thought possible given his environmental exposure history. Following the bacterial culture reports and the antibiotic sensitivity patterns, a diagnosis of melioidosis was made and the antibiotics were changed to intravenous Meropenem 1 g 8 hourly and Metronidazole 500 mg 8 hourly. Metronidazole was continued due to the presence of necrotic tissue and suspicion of superadded anaerobic infection. However, after 1 week he clinically deteriorated with worsening wound infection, fever and septic shock. He was resuscitated with fluids and commenced on noradrenaline. He required inotropic support for 4 days. He underwent another wound debridement and the dose of intravenous Meropenem was escalated to 2 g 8 hourly while continuing intravenous Metronidazole 500 mg 8 hourly and oral Co-trimoxazole 1920 mg 12 hourly was also added. He improved after a few days following the second wound debridement and supportive management. Fortunately, he did not develop any other organ dysfunction. All three antibiotics were continued for a period of 8 weeks.

He was screened for other foci for infection with chest radiograph, transthoracic and trans-oesophageal echocardiogram, abdominal ultrasonography and urine culture and all were negative. A computed tomography of the chest and abdomen was not performed as he did not have any clinical features to suggest a chest or abdominal foci of infection and the basic imaging were negative.

His temperature normalised 4 days after the second wound debridement and escalation of antibiotic therapy. However, he further required repeated wound debridement and removal of necrotic and infected tissue. His inflammatory markers became normal after 5 weeks of antibiotics (C-reactive protein of 2.6 mg/dl). Wound healing with secondary intention was achieved after 6 weeks (Fig. 1). He was given a total course of intravenous antibiotics for 8 weeks and was discharged on oral co-trimoxazole 1920 mg 12 hourly. Repeat X-rays at 2 months showed features of chronic osteomyelitis (Fig. 1). Due to the high risk of recurrence, the eradication phase was continued for a period of 6 months.

At 6 months follow up, he had satisfactory functional outcome with acceptable range of motion. There was no clinical evidence of relapse. However, the plain radiographic changes were persistent without any worsening.

## Discussion and conclusions

Melioidosis was first reported in Burma in 1912 and is now endemic across the tropical countries, specifically in Southeast Asia and Northern Australia [3, 5]. Diabetes mellitus is identified as a major risk factor and therefore, the increasing pandemic of diabetes has been postulated as a probable cause for the increasing prevalence and severity of melioidosis [3]. The facultative intracellular lifestyle and the virulence factors of the bacterium allow survival within various types of tissues by resisting host immune responses. The presentation and the prognosis depend on the route of entry and the type of tissue infected and also the host immunity and antibiotic sensitivity [3]. Diagnosis requires a high index of clinical suspicion and is confirmed by microbiological culture or serological evidence in the presence of a compatible clinical presentation. Delayed recognition of the disease is associated with poor clinical outcomes [3].

Musculoskeletal melioidosis is infrequently seen [1, 4]. A systematic review of cases of human melioidosis from 1990 to 2015 showed that 805 (8.2%) of 9833 patients had musculoskeletal infection [6]. They usually manifest as osteomyelitis with surrounding soft tissue infection and rarely, septic arthritis [4, 6]. The knee joint is the commonest joint affected in melioidosis and other joints include hip, shoulder and ankle [1, 4]. The disease may affect all age groups, but the peak rate is seen among the 40 to 60-year age group [1, 4]. A retrospective analysis demonstrated that there is a considerable risk (27.5%) of osteomyelitis of the adjacent bones in melioidosis associated septic arthritis [7]. Bone involvement was associated with repeated surgeries, prolonged hospital stay and recurrence. Those with musculoskeletal melioidosis may have multifocal disease involving adjacent or distant bone segments and joints and were associated with poorer outcomes [7].

In musculoskeletal melioidosis, haematogenous spread following percutaneous inoculation is postulated as the major mode of transmission [4]. However, the bacterium may also reach by direct spread from adjacent organs and soft tissue [4]. In our patient, local percutaneous inoculation may be possible due to his occupation. However, he denied any recent history of trauma or wounds at the onset of the illness. Early surgical debridement and drainage with appropriate administration of intravenous antibiotics is the cornerstone of management [1, 4]. A prolonged course of intravenous antibiotics is often needed due to the slow resolution of the infection [1, 4].

The treatment of melioidosis involving the musculoskeletal system is similar to the standard therapy. It includes an initial intensive phase, eradication therapy, surgical debridement and adjuvant therapy where relevant [3, 8]. In the initial intensive phase, intravenous ceftazidime or meropenem is given for 10–14 days. Observational data support the use of meropenem as the drug of choice for critically ill patients (similar to the reported case) with severe melioidosis [3, 8]. A prolonged intensive therapy may be considered for critically ill patients, osteomyelitis and septic arthritis. The therapeutic response may be slow and variable depending on the type and site of infection [3, 8].

After the completion of intensive therapy, eradication therapy with oral antibiotics for 3–6 months is essential to prevent recrudescence and relapse [3, 8]. Trimethoprim–sulfamethoxazole is used as the preferred choice for eradication therapy and doxycycline and co-amoxiclav are second-line choices depending on the sensitivity pattern of the organism, allergy history of the patient and adverse effects experienced [3].

Duration of treatment in each phase is decided based on the severity and the clinical response [3, 8, 9]. Prolonged intensive therapy for critically ill patients with melioidosis has lowered mortality. A retrospective analysis of melioidosis patients by Pitman et al. has shown that a median duration of 4 weeks was given for the initial intensive therapy and only 5 patients (1.2%) experienced relapse [9].

Surgical treatment is commonly required in musculoskeletal melioidosis [1, 3]. Large abscesses in muscles require incision and drainage with adequate debridement. Septic arthritis usually requires an arthrotomy and washout. Osteomyelitis may be extensive especially when the treatment is delayed and may often require repeated debridement of the necrotic bone segments [1, 3].

Adjuvant immune-modulating therapy may be considered in selected patients as immune function plays a crucial role in the pathogenesis. Critically ill patients may benefit from novel immune-modulating therapies, such as granulocyte-macrophage colony-stimulating factor, interleukin-7 and anti-programmed cell death protein 1 [3]. However, the evidence is not strong enough to support routine use and further studies are necessary [3].

Melioidosis associated peri-prosthetic infection is extremely rare and only one previous case has been reported [5]. Lin et al. reported a patient who developed melioidosis associated septic arthritis of the knee joint after total knee replacement. The patient needed repeat surgical interventions and finally the removal of the prosthesis with a prolonged course of antibiotics [5]. Our patient developed melioidosis associated osteomyelitis and septic arthritis of the ankle joint following a medial malleolar internal fixation after a period of 10 years. To date, such a presentation has not been reported. Long-standing poorly controlled diabetes and exposure to mud are the risk factors in our patient. He required removal of the prosthesis with repeated wound debridement and prolonged course of intravenous antibiotics. Furthermore, higher dose of meropenem was administered considering the worsening of wound infection, clinical deterioration and retained prosthesis. The local protocol of the hospital microbiologists is to increase the dose of meropenem in severe non-responding infections. Although this is not compatible with the current guidelines, we believe that this was necessary to manage this patient with severe wound infection associated with the retained prosthesis. The intravenous metronidazole was also continued up to the same period due to the initial suspicion of an anaerobic infection due to mud exposure.

Melioidosis associated peri-prosthetic infection is extremely rare. We report a unique presentation of osteomyelitis and septic arthritis of the ankle joint following medial malleolar internal fixation. The diagnosis of melioidosis should be considered in peri-prosthetic infections, in susceptible individuals. Proper surgical debridement and retrieval of material for microbial cultures should not be delayed. A high index of suspicion is needed in susceptible individuals for accurate diagnosis. Commencement of intravenous antibiotics with proper wound debridement and removal of prosthesis where relevant are essential in the management.

## Data Availability

All data generated or analysed during this study are included in this published article.

## Abbreviations

**HbA1c**: **Haemoglobin A1c**
